# Case Report: Nasal Cavity Epithelial-Myoepithelial Carcinoma With High Fluoro-D-Glucose Uptake on Positron Emission Tomography/Computed Tomography

**DOI:** 10.3389/fmed.2021.664520

**Published:** 2021-12-21

**Authors:** Jacques Dzuko Kamga, Jean-Christophe Leclere, Arnaud Uguen, Karim Amrane, Ronan Abgral

**Affiliations:** ^1^Department of Nuclear Medicine, University Hospital of Brest, Brest, France; ^2^Department of Otorhinolaryngology, University Hospital of Brest, Brest, France; ^3^Department of Pathology, University Hospital of Brest, Brest, France; ^4^EA 3878 GETBO, IFR 148, Bretagne Loire University, Brest, France

**Keywords:** nasal cavity, high grade, high avidity, ^18^FDG-PET/CT, epithelial-myoepithelial carcinoma (EMC)

## Abstract

Epithelial-myoepithelial carcinoma (EMC) is a rare malignant neoplasm arising most frequently in the salivary glands and exceptionally in the nasal cavity. EMC accounts for ~1–2% of salivary gland tumors. Even if the nodal and distant metastasis rates are low, tumor staging remains indicated. Here, the authors present the 2-deoxy-2-[^18^F]fluoro-D-glucose PET-CT (^18^F-FDG-PET/CT) study of a very rare case of biopsy-proven EMC of the left nasal cavity. This ^18^F-FDG-PET/CT was performed to stage this tumor and guide the therapeutic strategy due to an atypical high-grade presentation in immunohistochemistry. To our knowledge, this is the first case reporting such high ^18^F-FDG avidity of EMC of the nasal cavity in PET/CT.

## Introduction

Epithelial-myoepithelial carcinoma (EMC) is a rare malignant tumor, accounting for ~1% of all salivary gland tumors (1); it is localized mainly in the parotid and submaxillary glands (2). This carcinoma also rarely occurs in the nasal cavity (3). EMC is considered as a low to intermediate grade malignancy and is associated with 5- and 10-year overall survival rates of respectively 72.7 and 59.5% (4). Regional lymph node invasion and distant metastases are rare and occur in less than 5% of cases (4).

To date, 2-deoxy-2-[^18^F]fluoro-D-glucose (F-FDG) PET-CT (^18^F-FDG-PET/CT) is not recommended for the characterization of salivary gland tumors (differentiation between benign and malignant disease) but can be proposed for the staging assessment of histologically proven malignancy (5).

We report a very rare case of biopsy-proven high-grade EMC of the left nasal cavity with high ^18^F-FDG avidity in PET/CT.

## Case Description

A 66-year-old man without previous medical history was referred to our department to undergo a pre-therapeutic ^18^F-FDG-PET/CT for the staging of an EMC of the left nasal cavity discovered on the symptomatology of epistaxis. ^18^F-FDG PET/CT was performed on a digital Biograph Vision 600 (Siemens Healthineers, Knoxville, TN, United States). The patient fasted 6 h before the intravenous injection of ~3 MBq/kg of ^18^F-FDG. Following the injection, the patient remained in a quiet room for approximately 60 min before acquisition and was then scanned from the top of the skull to the mid-thigh in the arms-down position (6).

Free breath acquisitions were performed for both PET and CT. The PET data were acquired in three-dimensional (3D) continuous bed motion mode. Different scan speeds were set (3 mm s^−1^ over the head, 2 mm s^−1^ over the lungs, 1.4 mm s^−1^ over the abdominopelvic region, and 5 mm s^−1^ over the legs). The PET data were reconstructed thanks to ordered subsets expectation-maximization algorithm, using CT based attenuation correction, Compton scattering correction, resolution modeling, and time of flight correction (True X PSF+TOF OSEM3D), with 3 iterations, 5 subsets, and a 2 mm Gaussian filter, into a 440 × 440 matrix corresponding to 1.65 × 1.65 × 1.65 mm voxels.

**Figure 1:**
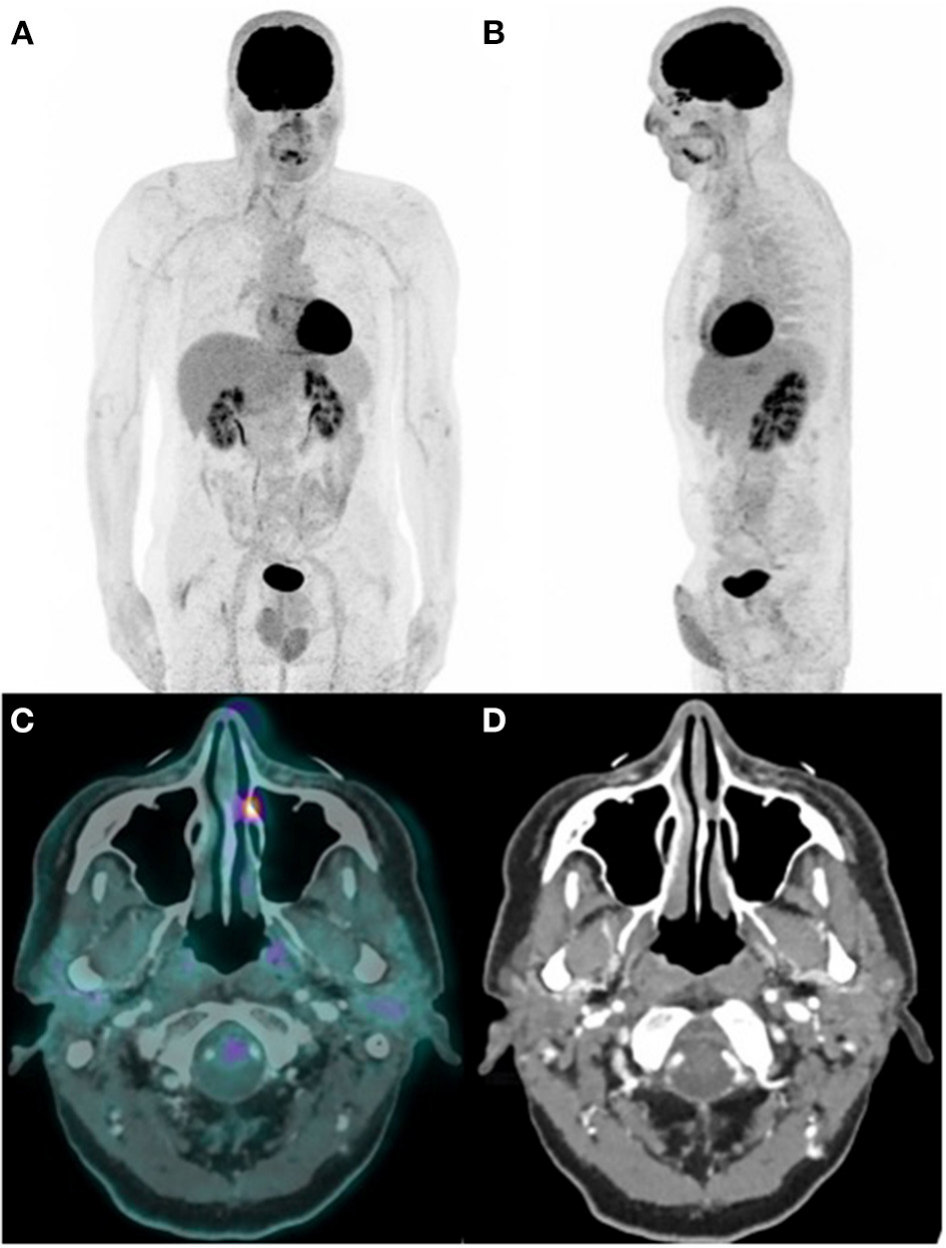
Fluoro-D-glucose PET/CT (FDG-PET/CT) images. **(A,B)** PET MIP **(C)** fused PET/CT, and **(D)** CT.

The ^18^F-FDG-PET (Figure 1) revealed no locoregional or distant remote extension (Figures 1A,B) but showed a high tracer uptake (SUVmax = 8.9) matching with a tissue density lesion of the left nasal cavity area on the fused CT (Figures 1C,D), reflecting a significant metabolic activity.

**Figure 2:**
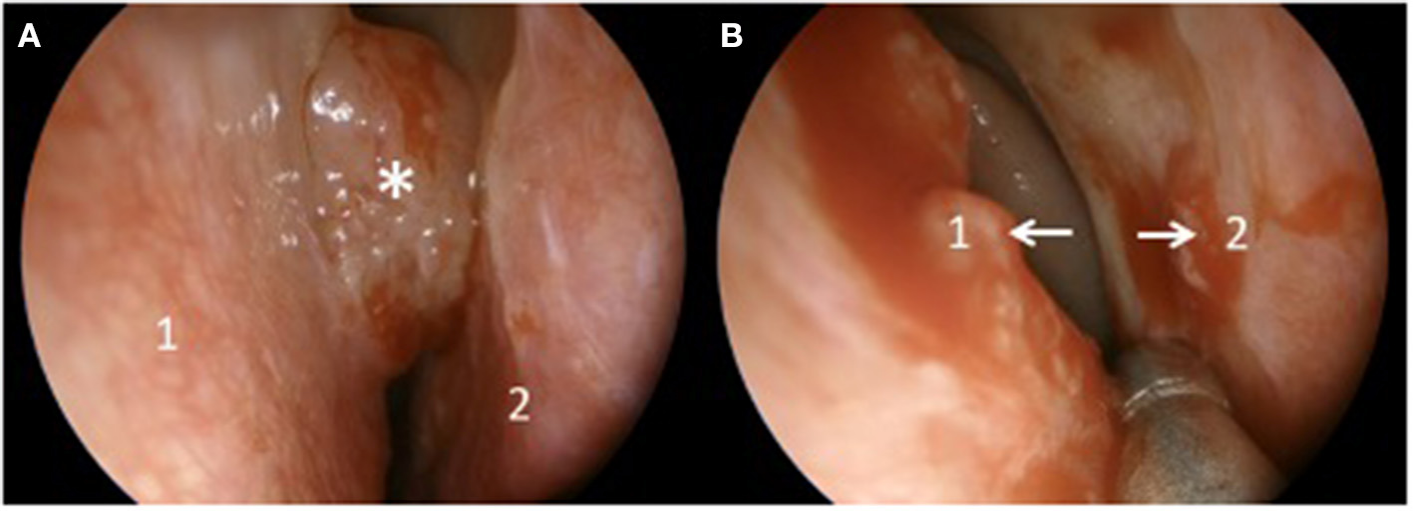
Endoscopic examination before **(A)** and after **(B)** tumor debulking.

The left nasal cavity EMC was removed by endoscopic resection [Figure 2 showed the nasal septum (Figure 2A1), the lower left nasal turbinate (Figure 2A2), and the tumor (Figure 2A^*^)], and after debulking, the origins of the tumor were identified (Figure 2B white arrows) before proceeding with a septectomy (Figure 2B1) and a left medial maxillectomy (Figure 2B2) to obtain a complete resection with safety margins of 1 cm.

**Figure 3:**
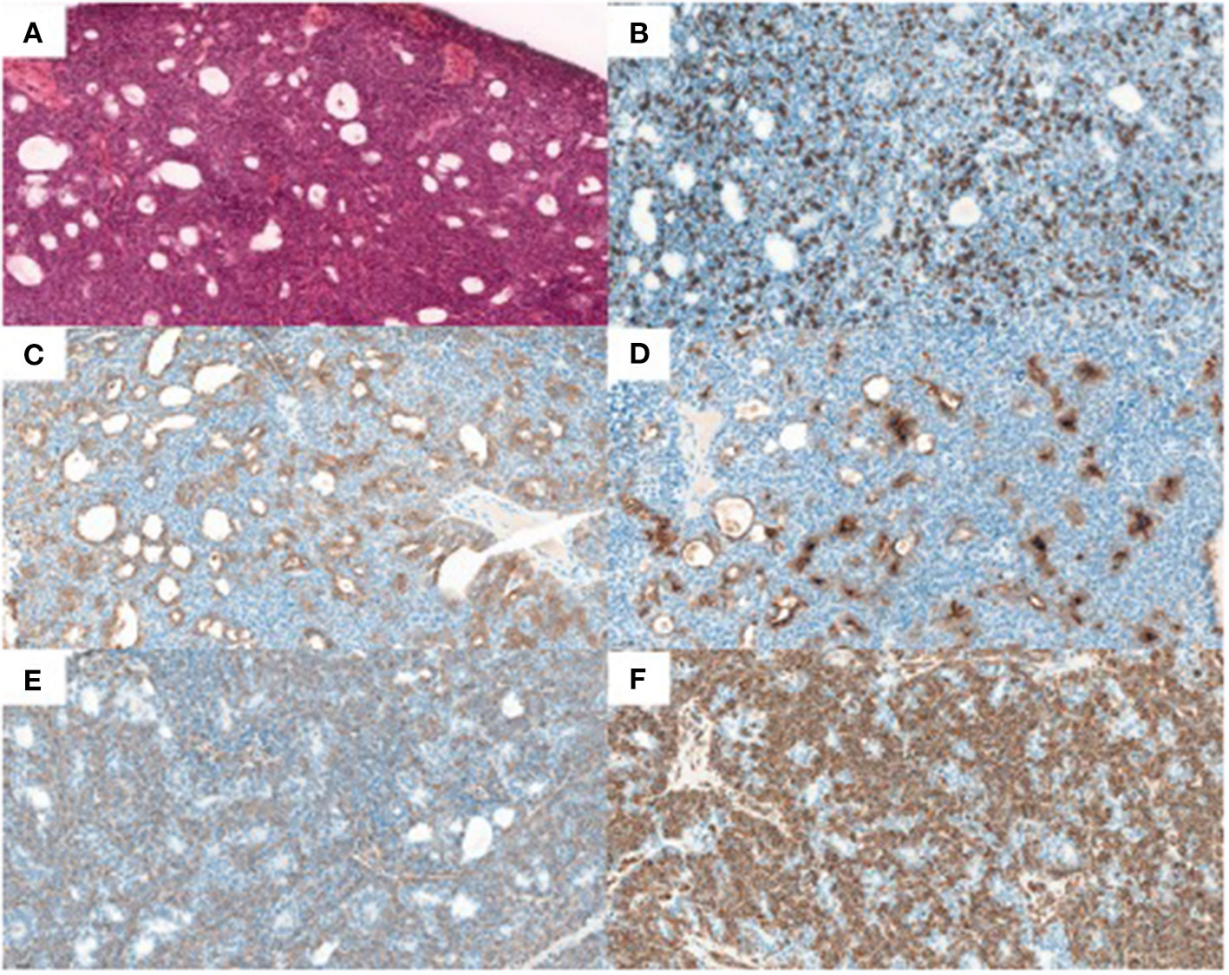
Histopathology results. **(A)** hematoxylin-eosin-saffron, x20, **(B)** Ki-67 MIB1, x20, **(C)** pan-keratin AE1/AE3, x20, **(D)** epithelial membran antigen-EMA, x20, **(E)** smooth muscle actin, x20, and **(F)** vimentin, x20.

The histopathology analysis (Figure 3) of the resected tumor showed a biphasic tumor with small luminal ducts surrounded by myoepithelial abluminal cells (Figure 3A: hematoxylin-eosin-saffron, ×20) (7). The ki-67 immunohistochemistry proliferative index was about 50% (Figure 3B: MIB1, ×20). The immunohistochemistry analysis showed the epithelial phenotype of luminal cells (Figure 3C: pan-keratin AE1/AE3, Figure 3D: epithelial membrane antigen-EMA, ×20) and the myoepithelial phenotype of abluminal cells (Figure 3E: smooth muscle actin, Figure 3F: vimentin, ×20).

## Discussion

The reported tumor was located in the region of the inter-sinus maxillary septa and connected to the nasal septa by a tissue extension partitioning the left nasal cavity that corresponds to a rare presentation. Indeed, in a recent large population-based analysis, Gore et al. reported 468 cases of EMC in the United States from 1973 to 2014 and identified only six cases (1.3%) of tumors in the nasal cavity (4).

We found an atypical high ^18^F-FDG uptake (SUVmax = 8.9) over this lesion as EMC has been reported to show no significant ^18^F-FDG uptake on PET/CT which may be associated with its very common low-grade malignant potential (5, 8).

To our knowledge, this is the first case of EMC of the nasal cavity presenting such ^18^F-FDG avidity on PET/CT (8–10). Only Sharma et al. reported a case of ^18^F-FDG -positive EMC of the lacrimal sac incidentally highlighted on a PET/CT performed in a patient with colorectal cancer (11). Nevertheless, this ^18^F-FDG avidity could be explained by the high Ki-67 expression (50%) that was found in the immunohistochemistry analysis, classifying this tumor atypically in high grade. Ki-67 is a protein expressed in all phases of the cell cycle, except the G0-phase, therefore estimating the fraction of proliferative cells in tissues (12). Furthermore, the Ki-67 index and SUVmax value are well-known to be highly correlated in other head and neck cancers (13). Another approach based on sequential biphasic ^18^F-FDG-PET scanning protocol could have been used to distinguish benign and malignant lesions (14, 15).

Finally, although this presentation remains extremely rare, it probably increases the risk of locoregional or distant remote extension (16).

## Conclusion

2-deoxy-2-[18F]fluoro-D-glucose PET-CT (FDG-PET/CT) might be an interesting tool for assessing remote extension of such rare presentation of EMC with high Ki-67 expression.

## Data Availability Statement

The raw data supporting the conclusions of this article will be made available by the authors, without undue reservation.

## Ethics Statement

All procedures performed in this study were in accordance with the Ethical Standards of the Institutional Research Committee on Human Experimentation and with the Helsinki Declaration of 1975, as revised in 2008. Ethical review and approval were not required in accordance with the national and institutional requirements. The patient provided written informed consent.

## Author Contributions

RA and JDK are the guarantors of the manuscript and analyzed the imaging. KA and J-CL ensured the clinical follow-up of the patient. AU provided the histopathology. JDK, J-CL, AU, KA, and RA contributed to drawing up. All authors contributed to the article and approved the submitted version.

## Conflict of Interest

The authors declare that the research was conducted in the absence of any commercial or financial relationships that could be construed as a potential conflict of interest.

## Publisher's Note

All claims expressed in this article are solely those of the authors and do not necessarily represent those of their affiliated organizations, or those of the publisher, the editors and the reviewers. Any product that may be evaluated in this article, or claim that may be made by its manufacturer, is not guaranteed or endorsed by the publisher.
